# Consequences of the Corona crisis on outpatient oncological care – a qualitative study among nurses and medical assistants

**DOI:** 10.1371/journal.pone.0276573

**Published:** 2022-10-21

**Authors:** Ulrich Kaiser, Ursula Vehling-Kaiser, Jörg Schmidt, Ana Hoffmann, Florian Kaiser

**Affiliations:** 1 Clinic and Polyclinic for Internal Medicine III, Regensburg University Hospital, Regensburg, Bavaria, Germany; 2 Oncological-Palliative Network Landshut, Landshut, Bavaria, Germany; 3 Institute for Market Research in the Health Care System Munich, Munich, Bavaria, Germany; 4 VK&K Studien GbR Landshut, Landshut, Bavaria, Germany; 5 Clinic for Hematology and Medical Oncology, University Medical Centre Göttingen, Göttingen, Lower Saxon, Germany; Waikato Institute of Technology, NEW ZEALAND

## Abstract

**Introduction:**

The Covid-19 pandemic has caused great personal stress for medical staff. To ensure adequate outpatient care for cancer patients, extensive safety and hygiene measures must be taken. This interview-based study examines the effects–both personal and professional–of the pandemic on the work routine of outpatient hematology/oncology nurses and medical assistants.

**Patients, materials and methods:**

Half a year after the outbreak of Covid-19 and the introduction of infection control regulations in three outpatient hematological/oncological centers, the affected medical staff (n = 15) were surveyed about the consequences for patient care and clinical work using audio-recorded telephone interviews. The interviews were transcribed and analyzed using a qualitative content analysis.

**Results:**

The Covid-19 pandemic has complicated the medical care of cancer patients, but only a slight deterioration of medical and psycho-oncological care was observed. The level of stress experienced by medical staff is moderate, with hygiene and safety measures at the workplace helping to reduce stress.

**Conclusion:**

From the point of view of medical staff, the Covid-19 pandemic has had a moderate impact on the outpatient care of cancer patients. Safety measures against Covid-19 are decisive for ensuring the continuation of therapy and for motivating employees.

## Introduction

At the end of 2019, severe respiratory infections caused by the RNA beta coronavirus SARS-CoV-2 appeared in China for the first time [1, 2]. This was followed by a rapid worldwide spread of the coronavirus disease (Covid-19). An initial nationwide lockdown with strict rules around personal interaction and hygiene was initially effective in curbing the number of cases in Germany, whereas a loosening of the regulations around personal contacts led to a renewed increase in infections [1]. Covid-19 can have varying clinical manifestations in terms of severity, causing death in severe cases [1, 3]. People with a weakened immune system or suffering from a malignant disease are particularly at risk [1, 4]. To guarantee high-quality medical care for hematological/oncological patients, structural and organizational adjustments had to be made for outpatient and inpatient wards. The German Society for Hematology and Oncology (DGHO) and the European Society for Medical Oncology (ESMO) issued a series of hygiene and safety recommendations, which place special emphasis on the necessity of using telemedicine or digital medicine [4, 5].

The Covid-19 pandemic has led to clear signs of stress and uncertainty among medical staff: the risk of delaying tumor therapy/treatment must be weighed against the infection risk of Covid-19 in severely immunocompromised patients [4–8]. Close contact with patients results in an increased risk of infection for medical professionals [9]. Stress is often compounded by a lack of protective equipment–particularly in the outpatient wards–or restrictive changes to the work routine [10, 11]. Women and nurses in particular seem to be affected [10, 12]. During the pandemic, stress relief through family and social networks has been limited by contact restrictions [9]. Through all these factors in combination, the Covid-19 pandemic has caused a new, immense stress among medical staff [9–14]. Informing staff properly and providing personal protection equipment and psychological support are recommended to reduce stress [9, 11, 13, 15, 16].

Many studies on this topic have been conducted within inpatient settings, but similar studies on outpatient care have been quite rare [13]. The present study deals with problems and experiences in the context of the Covid-19 pandemic from the perspective of medical staff (medical assistants and nurses) who work in outpatient hematological/oncological care. The resulting personal and professional consequences and the effects on outpatient care were examined by means of audio-recorded phone interviews.

## Materials and methods

### Aim, design and setting

At the beginning of the Covid-19 pandemic, identical hygiene and safety measures were introduced in three hematological/oncological outpatient clinics in Bavaria, the largest state in south east Germany. These included a two-shift work system, entry checkpoints, no entry for companions, distancing rules, personal hygiene rules, compulsory face masks (FFP2), regular surface disinfection and ventilation, shortened consultation times and implementation of digital consultations [4, 5]. When the study was conducted, vaccines against Covid-19 were not available and testing for Covid-19 was not yet introduced nationwide.

All hematological and oncological diseases that can be handled on an outpatient basis are treated in the participating practices. This includes, for example, antiproliferative systemic therapies (except stem cell transplantation), palliative care and psycho-oncological care, bone marrow an ascites punctures or ultrasound examinations. All patients have a statutory or private health insurance. There are no additional treatment costs for patients.

The participating outpatient clinics are approved institutions of the German healthcare system and certified by the German Caner Society, the German Society for Hematology and Oncology and the European Society for Medical Oncology; national and international standards and guidelines are used to ensure adequate medical and psycho- oncological care.

The aim of the qualitative survey was to get more insights into the following problems:

- Influence of the Covid-19 pandemic on outpatient (nursing) care for cancer patients
  ○ Changed interactions
  ○ Perceived fears
  ○ Influence on the mutual trust between medical staff and cancer patients
  ○ Changed medical/psycho-oncological care
  ○ Influence on the course of the disease
- Influence of the Covid-19 pandemic on (nursing) work in outpatient oncology
  ○ Effects on the clinical environment
  ○ Effects on workflows
  ○ Personal stress and possible solutions
- Importance of new technology (telemedicine, digital medicine)

Audio-recorded, semi-structured, qualitative interviews via telephone were used as the survey method. Thanks to the open form, which invites conversation, this qualitative method ensures an atmosphere where the interviewees can express any relevant opinions and thoughts relating to the topic, providing meaningful results typical of the respective target group [17–20]. In addition, the interviewer can ask directly if something is unclear, or elicit further important information about the topic by using supplementary questions. The interviews were conducted in German. To guarantee an unbiased interview situation, the participants had no access to structured patient feedback.

### Participants and data collection

At the end of May 2020 –almost four months after the above-mentioned measures were introduced–all nurses (n = 6) and medical assistants (n = 20) from the participating institutions were invited without exception to take part in qualitative, guided telephone interviews. Due to the small sample size (n = 26), there were no further selection or saturation criteria for potential participants. In the participating outpatient clinics, patient care is provided exclusively by physicians, nurses and medical assistants. Other professional staff is not involved. Physicians were excluded from the study, because nurses and medical assistants have more intensive and closer patient contact. Different perspectives on the safety measures and dealing with the Covid-19 pandemic can be avoided. Therefore, further sampling methods were not necessary.

A semi-structured interview guide consisting of twenty-five open-ended questions (Table 1) had been developed for this study. The average interview was 30 minutes long. There were no time restrictions; the respondents had sufficient time to give their answers. The interviews were conducted in June and July 2020 by the Institute for Market Research in Health Care (S1 Appendix).

**Table 1:** Interview guide for nurses and medical assistants.

**Introductory questions**
1. What do you think of spontaneously when you hear the words corona pandemic?
2. How are you personally coping with the Covid-19 pandemic?
3. What worries you most about the Covid-19 pandemic?
**Impact of the Covid-19 pandemic on patient care**
4. How does the Covid-19 pandemic affect patient care?
5. In concrete terms, how has the treatment of patients changed as a result of the pandemic?
6. Which changes–in relation to your patients–do you think are the most serious as a result of the pandemic?
7. Has the Covid-19 pandemic–in your experience–changed patients’ attitudes towards their tumor disease? (e.g. the duration of therapy; therapy termination or postponement of therapies)
8. What fears do your patients primarily have?
9. How do you deal with patients’ fears during treatment?
10. Do you think that patient care has deteriorated from a medical point of view?
11. Do you think that patient care has deteriorated from a psycho-oncological point of view?
12. In your experience, does the Covid-19 pandemic have an impact on the relationship of trust between medical staff and patients? If so, in what ways?
13. Do you think the Covid-19 pandemic could affect the disease progression of your patients? If so, how?
14. Do you have the impression that patients have changed their statements about Covid-19 over time, i.e. have they withheld information about possible Covid-19 infections concerning themselves or those in their surroundings, e.g. for fear of treatment delays or for other reasons?
**Influence of the Covid-19 pandemic on clinical work**
15. How does the Covid-19 pandemic affect the clinical environment?
16. How is or has been the relationship with doctors and medical staff (colleagues) during the Covid-19 pandemic?
17. Do you feel safe in the clinic you work in?
18. If so, what gives you this feeling of safety?
19. If not, what would give you a sense of safety in the clinic?
20. To what extent have the processes in oncological practice changed for you?
21. If so, are these changes a burden for you?
22. How much personal pressure do you feel you are under from the Covid-19 pandemic and how is this reflected?
**Closing questions**
23. What is particularly important for you personally during the Covid-19 pandemic when you arrive at the clinic / your workplace?
24. Do you consider the communication options offered during the Covid-19 pandemic (telephone consultation, video consultation) to be sufficient for patients?
25. If not, what is missing?

### Data analysis

The interviews were transcribed for further processing. The answers were analyzed using the qualitative content analysis method according to Mayring [20, 21]. This is a multi-stage, structured, reproducible analytical process whose steps include categorization, coding, re-evaluation and analysis of the interviews, thereby abstracting qualitative data systematically and transparently. After an initial review of the material, the interview questions were taken as the main categories. Subsequently, subcategories were formed for each main category by summarizing and identifying key topics. All the interview data were then sorted using the system of categories, and documented in tabular form (coding). In the process, the categories were constantly re-evaluated and adapted as new insights were obtained. Finally, the results were summarized and analyzed. In addition, literal statements (quotes) by the interviewees were used in the analysis in anonymized form, which highlighted the participants’ ways of thinking and expressing themselves. To ensure interpersonal validity, four researchers conducted and discussed the analytical process. Additionally, to highlight special aspects of the results and according to Mayring [20], we conducted quantitative data analysis (as part of the qualitative analysis) of the final qualitativ interview material.

### Ethical considerations

After submission of a proposal for the present study to the Ethics Committee of the Bavarian Medical Association in Munich, no ethics vote was required, because the study was classified as quality control. The interviews took place only after the respective participants had been informed by the study physicians/staff and had given their written informed consent, that was collected at the study center. All statements were anonymously documented via audio recording.

## Results

15 employees (5 nurses and 10 medical assistants) agreed to participate in the survey. All 15 participants were female and the majority were under 50 years old (20–29 years: n = 7, 30–39 years: n = 1, 40–49 years: n = 3, 50–59 years: n = 3, no information: n = 1). Their professional experience ranged from 1 year to 40 years (1–5 years: n = 4, 6–10 years: n = 2, 11–15 years: n = 1, 16–20 years: n = 1, 21–25 years: n = 3, 26–30 years: n = 2, 31–40 years: n = 2). All nurses and medical assistants surveyed were actively and personally involved in the outpatient care of hematological/oncological patients.

### Influence of the Covid-19 pandemic on outpatient care of cancer patients

#### Complicated care of cancer patients

14 out of 15 respondents reported the Covid-19 pandemic has had a major influence on patient care. In particular, the new safety and hygiene regulations complicate every day work. In addition, patients’ fear of infections was felt to have resulted in delays in treatment.

Quote: *“Patients are questioned, have to keep their distance, take their temperature. All these are combined to impact the clinical routine.*”

Certain restrictions such as bringing companions, physical distancing and compulsory face masks hinder adequate delivery of counseling and support for cancer patients.

Quote: *“Face masks make contact with the patient more difficult.*”

#### Cancer patients’ increased fears around Covid-19

About half of the participants (8 out of 15) observed a change in their patients’ attitude towards their tumor disease. Due to fear of being infected with Covid-19, therapy was postponed/discontinued by some patients. According to the respondents, the fear of becoming infected with COVID-19 is the top priority for cancer patients, rather than their worries about their tumor disease or isolation/loneliness.

Quote: *“Patients have a fear of infection because their immune system is shut down by chemotherapy.*”

However, 7 out of 15 respondents see no change in the attitude of cancer patients towards their disease. Antiproliferative therapies are still used by many patients.

All participants tried to alleviate or reduce patients’ fears through intensive discussions of the current problems as well as by providing information about the safety measures introduced at the clinic.

### Changed relationship of trust between medical staff and cancer patients

The respondents expressed different opinions on the influence of Covid-19 on the relationship of trust between medical staff and patients. While 9 of the participants saw no difference, 6 reported both positive and negative changes. An increase in trust and a need to talk more to the medical staff was reported as a positive change, leading to a stronger relationship between nurses/medical assistants and patients. Some of the interviewees were not pleased with misleading answers given by patients at the entrance to the clinic and during security checks, where information was withheld or given incorrectly on purpose.

Quote: *“She kept silent that a family member was positive and she was also tested, which I find unfair to the others.*”

However, some respondents also expressed understanding for these patients and their concern about a possible delay of their therapy.

#### Hardly any deterioration in medical and psycho-oncological care, but an overall negative influence of Covid-19 on disease progression in cancer patients

The majority of respondents saw no deterioration in medical (12 out of 15) or psycho-oncological (10 out of 15) care as a result of the Covid-19 pandemic: medical and psycho-oncological care was still ensured by the new security measures and digital consultations. The reasons for a possible decrease in the quality of medical care were shorter patient-doctor interactions, postponement or cancellations of appointments resulting from patient anxiety or from safety measures. The reasons for a possible reduction in psycho-oncological care were isolation and loneliness and limited opportunities for consultations due to the safety measures (short consultations, masks, distancing).

Regardless of patient care, some of the interviewees (7 out of 15) thought that the Covid-19 pandemic would have a negative impact on the disease progression in cancer patients. The reasons cited were psychological stress and a delay in diagnostic and therapeutic measures.

### Influence of the Covid-19 pandemic on work in outpatient oncology

#### Hardly any negative effects on the clinical environment

There was a mixed response about pandemic´s effect on working environment. 6 felt there was no major effect and 4 felt there were positive effects of the pandemic on their working environment.

Quote: *“In my opinion, positive effects. From the point of view of the employees, it allows for closer collaboration, cohesion and more intensive cooperation.*”

Especially the closer collaboration and solidarity amongst medical staff was emphasized. The relationships with other team members hadn’t deteriorated for any of the participants.

The shift system and its consequences (changed working hours, time pressure, negative hours) were cited by five respondents as the main problems causing a bad clinical environment.

Quote: *“Rather negative, because we have introduced shifts for medical assistants.*”

### New protection and security measures are changing work processes

The most noticeable changes in the clinic processes were the introduction of shift work, distancing rules and security checks for patients. New hygiene rules, obligatory face masks and telephone consultations only played a minor role.

Quote: *“They have changed a lot. There used to be a hustle and bustle, now it has simply become more static.*”

For almost half of the respondents (n = 7), the Covid-19-related changes to their work routine were a cause of personal stress, primarily because the shift work or the changed working hours caused problems.

### The personal stress from Covid-19 is moderate

The biggest concerns about Covid-19 were the contact restrictions and the general risk of infection. Nevertheless, all participants were coping well with the Covid-19 pandemic; the situation was managed realistically and without fear.

Quote: *“Good. The restrictions bother me, but in the end, that’s the way it is, you have to adapt.*”

### Hygiene and safety measures in the workplace reduce the stress of medical staff

Accordingly, the participants’ personal stress caused by the Covid-19 pandemic is relatively moderate (Table 2; according to Mayring [20] quantitative data analysis can be part of qualitative analysis). The main reasons for stress were the consequences of the (general) isolation and safety measures.

**Table 2:** Personal stress suffered by the participants due to the Covid-19 pandemic.

	Medical staff (n = 15)
**Overview**	**n = 15**
High personal stress	4
Medium personal stress	3
Low personal stress	5
No personal burden	3
**Reasons for “high personal stress”**	**n = 4**
At first it was very difficult when you had to stay at home	1
Wearing mouth and nose protection all day is very stressful	1
Fear of infecting colleagues or the doctor in the clinic	1
Contact restrictions were very restrictive	1
**Reasons for “medium personal stress”**	**n = 3**
I would say medium personal stress, that is the hygiene measures and the time pressure	1
I love to travel and had many vacations booked	1
Uncertainty, can you do something, can you go on vacation	1
**Reasons for “low personal stress”**	**n = 5**
The greatest burden is the mouth and nose protection, otherwise no burden	1
You get stressed faster	1
My stress is limited	1
You change certain behaviors	1
Personal stress is not that high	1
**Reasons for “no personal burden”**	**n = 3**
No personal burden, I gladly avoid going to restaurants	1
At the moment I am not burdened at all	1
No burden, I didn’t think about getting sick	1

All 15 respondents felt safe working in the oncological outpatient clinics. This was primarily due to the protective equipment used and the strict security checks, besides the distancing rules, hygiene and safety measures and the sense of responsibility inspired by the clinics’ management.

The 3 most important things perceived as positive changes to the workplace were the protective equipment and obligatory face masks, the cleanliness and disinfection of the workplace and the compliance with hygiene regulations. The protection of colleagues and a good working atmosphere were also mentioned.

### Importance of new media (telemedicine, digital medicine)

#### New forms of communication make sense, but the personal contact is often missing

9 respondents considered telephone/video consultations to be a useful communication option in times of Covid-19, which should continue to be available after the pandemic has subsided.

Quote: *“I think it’s great under the circumstances and it could be maintained even after the pandemic has subsided.*”

Especially the presence of relatives (who were not allowed to enter the outpatient clinics due to security measures) during the telephone/video consultations and the lower stress levels for patients due to reduced trips into the outpatient clinics were perceived as positive.

Quote: *“Relatives can also listen during telephone consultations, which is very, very important.*”

However, the digital infrastructure (e.g., availability and use of digital media) should be improved in the future, especially for older people.

6 respondents find the telephone/video consultations critical, but they miss the personal touch.

Quote: *“No, it is a good alternative, but I think a telephone consultation can never replace a doctor/patient contact.*”

## Discussion

The Covid-19 pandemic has led to radical changes in the health care system in Germany [1], causing major professional and personal stress for medical staff [9–13, 22]. The imposed hygiene and safety measures resulted in massive changes to the work routine of oncological outpatient departments [4, 5]. The present interview-based study highlights the consequences of Covid-19 for the outpatient care of cancer patients as well as professional and personal consequences from the point of view of hematological/oncological medical staff (nurses and medical assistants).

8 out of 15 respondents observed an increased fear of being infected with Covid-19 in their cancer patients. From the point of view of the respondents, this led to increased postponements/discontinuations of tumor therapies. The fear of Covid-19 seemed to outweigh the fear of the tumor disease. This coincides with American and Dutch data which documented increased postponements or discontinuations of therapy of over 10% in oncological patients at the beginning of the Covid-19 pandemic [23, 24]. On the other hand, in the present study there was a slight decrease in follow-up examinations in the participating outpatient clinics, but–with the exception of the initial weeks of the pandemic–there was no decrease in the antiproliferative therapies carried out (2019: average of 532 antiproliferative therapies per 3 months; 2020: average of 543 antiproliferative therapies per 3 months). This coincides with the statements of other respondents (7 out of 15), who reported an increased fear of Covid-19, but no changes in the tumor therapies performed. Similarly, a survey of gynecological patients from May 2020 confirmed a desire among patients to continue tumor therapy despite the increased risk and fear of Covid-19 [25]. Overall, based on the results available, it could be argued that essential therapies are still being administered despite Covid-19, while non-essential (follow-up) examinations tend to be postponed. This is in line with the recommendations of the DGHO [4] and supported by the fact that a large number of respondents–despite the statements made above–reported no deterioration in medical (12 out of 15) or psycho-oncological (10 out of 15) patient care. From the point of view of nurses and medical assistants the care of tumor patients seems to be more difficult due to the Corona pandemic, but vital therapies can still be made possible by the new safety measures. The personal protective equipment in particular gives nurses and medical assistants a sense of security. So, their work in medical care can be continued despite Corona.

However, a reduction in personal contact with the patients resulting from the hygiene regulations was a cause of concern for the participants. In the field of hematology and oncology in particular, personal contact is conceived as an important cornerstone of the treatment concept. A decrease in personal contact due to the safety measures [23] could thus contribute to increased anxiety and a reduction of cancer patients’ quality of life [24]. Also, there seems to be an increased need among the patients, at least in some of them. Some of the respondents reported an increased trust and need for sharing on the part of the patients. On the other hand, the Covid-19 pandemic can also have a negative impact on the relationship of trust between medical staff and cancer patients. Some interviewees witnessed patients hiding their Covid-19 infection or typical symptoms during admission checks. In principle, understanding was expressed for the motives (fear of delaying therapy), but such behavior per se was regarded as incorrect behavior or even a breach of trust.

The negative impact of the Covid-19 pandemic on the mental health of medical staff has been demonstrated in several studies [9–13, 22]. However, in contrast to other studies reporting on the stress levels of staff, the present survey has revealed that medical staff is adapting relatively well to the new situation. This is remarkable insofar as the Covid-19 pandemic has exposed women and nurses to particularly high levels of psychological stress [10, 12] and the interviewees for this study were exclusively female. The fact that the respondents worked in outpatient hematology/oncology and not in an infection or intensive care unit may explain the relatively low stress levels. However, even in hematology/oncology, medical professionals often have close patient contact, exposing them to a corresponding risk of infection. The collaborative environment at the clinics might have had a positive effect, however. In times of a pandemic, the social environment plays a special role in reducing stress [9, 16]. Especially in times of general restrictions around personal contacts, a closer relationship with work colleagues could reduce psychological stress. Another important factor in stress reduction seems to be the safety measures taken to protect employees (and patients) [9, 11]. Not a single one of the participants felt exposed to a health (infection) risk at work and no one reported an avoidance of care. For all respondents, this was mainly due to personal protective equipment and the new safety and hygiene rules [9]. The concerns and personal stresses mentioned resulted from the risk of contagion, as well as from the consequences of individual isolation and safety measures. In particular, the newly introduced shift work was perceived as a significant source of stress, which also had a negative effect on the general mood in the workplace. Medical staff in German oncological outpatient clinics do not usually work in multiple shifts, so these changes have represented a significant intervention in the everyday life of the participants.

For the most part, the medical stuff surveyed consider the use of modern telecommunication technology (video/telephone consultations) during the Covid-19 pandemic to be a useful means of health-care delivery. They are a useful addition to short personal patient contacts without companions. This is also reflected in national/international data, which showed a massive increase in virtual health care of up to 1000% over the course of the Covid-19 pandemic [26, 27]. Hematological/oncological societies also recommend the use of telemedical measures to minimize contacts [4, 5]. However, some of the medical staff surveyed here do not see virtual consultations as an adequate substitute for personal contact. Digital tumor medicine is currently still in its infancy. A further evaluation of which patients can benefit from this in the long term, and in which situations, is certainly still required [27, 28].

### Limitations

The present work is based on qualitative interviews with medical assistants and nurses. The statements were not checked by quantitative analyses. Especially for the reported stress levels no standardized scales were used. Stress levels were self-perceived and can therefore be subjective. A precise quantitative verification was not possible. In addition, none of the respondents came from a hematological/oncological outpatient department with different safety precautions so that no comparative statements are available. Only 50% of the medical assistants took part in the study and all participants were female. A lack of information or differing perspectives (especially of male medical staff) cannot be excluded. However, all medical staff of the participant centers were female, so that there is probably no selection bias. The interviews took place when the Covid-19 infection rate was quite low. However, the statements, especially about the protective and security measures, are particularly important in times of increasing case numbers. The statements and conclusions must be viewed in conjunction with these aspects. The patient`s view on the consequences of the corona pandemic is not discussed in this paper. We did it in a separate work [29]. The survey was conducted in German. During the publication process there was a language shift into English. Thereby, contents of the questionary could have been lost or altered; but there is no impairment of the results.

### Conclusions

The Covid-19 pandemic has had a relevant impact on the outpatient care of tumor patients and on the work processes in hematological/oncological outpatient departments. However, from the point of view of nurses and medical assistants, medical care for malignant diseases appears to be only marginally impacted by the pandemic. The medical staff themselves are well able to deal with the threat of Covid-19 in their everyday work. Effective and noticeable protective and security measures can make a significant contribution to this.

## Supporting information

S1 Appendix Study framework planning. (PPTX)

S1 File (DOCX)

S2 File (PDF)
