# Supplementary material for: Consequences of the Corona crisis on outpatient oncological care – a qualitative study among nurses and medical assistants
Source: PLoS One. 2022 Oct 21;17(10):e0276573. doi: 10.1371/journal.pone.0276573 (PMC9586350; doi:10.1371/journal.pone.0276573)
Supplement: S1 Appendix — (PPTX) [file pone.0276573.s001.pptx]

## Slide 1
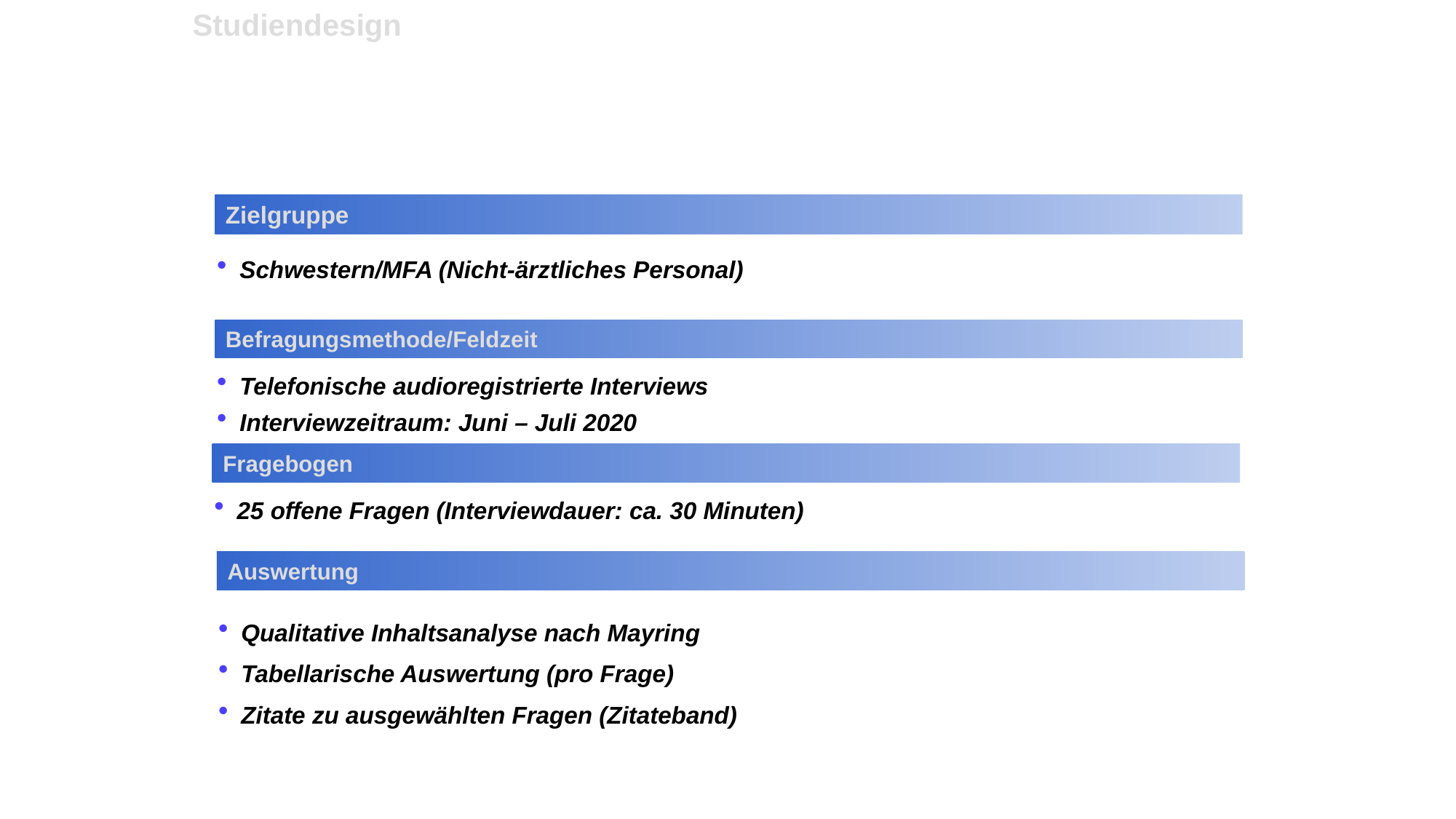

Studiendesign
Zielgruppe
Schwestern/MFA (Nicht-ärztliches Personal)
Befragungsmethode/Feldzeit
Telefonische audioregistrierte Interviews
Interviewzeitraum: Juni – Juli 2020
Fragebogen
25 offene Fragen (Interviewdauer: ca. 30 Minuten)
Auswertung
Qualitative Inhaltsanalyse nach Mayring
Tabellarische Auswertung (pro Frage)
Zitate zu ausgewählten Fragen (Zitateband)

## Slide 2
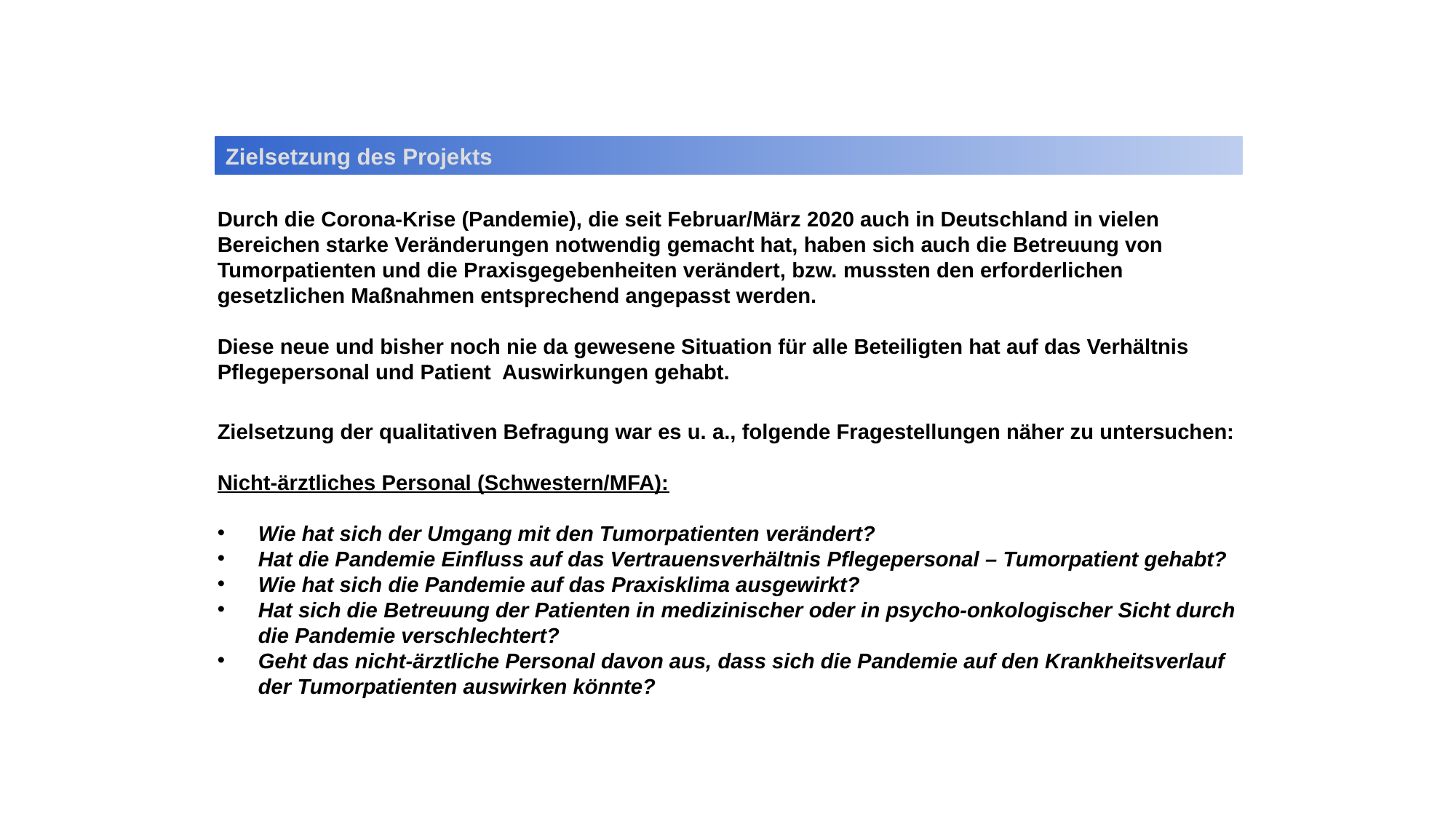

Zielsetzung des Projekts
Durch die Corona-Krise (Pandemie), die seit Februar/März 2020 auch in Deutschland in vielen Bereichen starke Veränderungen notwendig gemacht hat, haben sich auch die Betreuung von Tumorpatienten und die Praxisgegebenheiten verändert, bzw. mussten den erforderlichen gesetzlichen Maßnahmen entsprechend angepasst werden.
Diese neue und bisher noch nie da gewesene Situation für alle Beteiligten hat auf das Verhältnis Pflegepersonal und Patient Auswirkungen gehabt.
Zielsetzung der qualitativen Befragung war es u. a., folgende Fragestellungen näher zu untersuchen:
Nicht-ärztliches Personal (Schwestern/MFA):
Wie hat sich der Umgang mit den Tumorpatienten verändert?
Hat die Pandemie Einfluss auf das Vertrauensverhältnis Pflegepersonal – Tumorpatient gehabt?
Wie hat sich die Pandemie auf das Praxisklima ausgewirkt?
Hat sich die Betreuung der Patienten in medizinischer oder in psycho-onkologischer Sicht durch die Pandemie verschlechtert?
Geht das nicht-ärztliche Personal davon aus, dass sich die Pandemie auf den Krankheitsverlauf der Tumorpatienten auswirken könnte?

## Slide 3
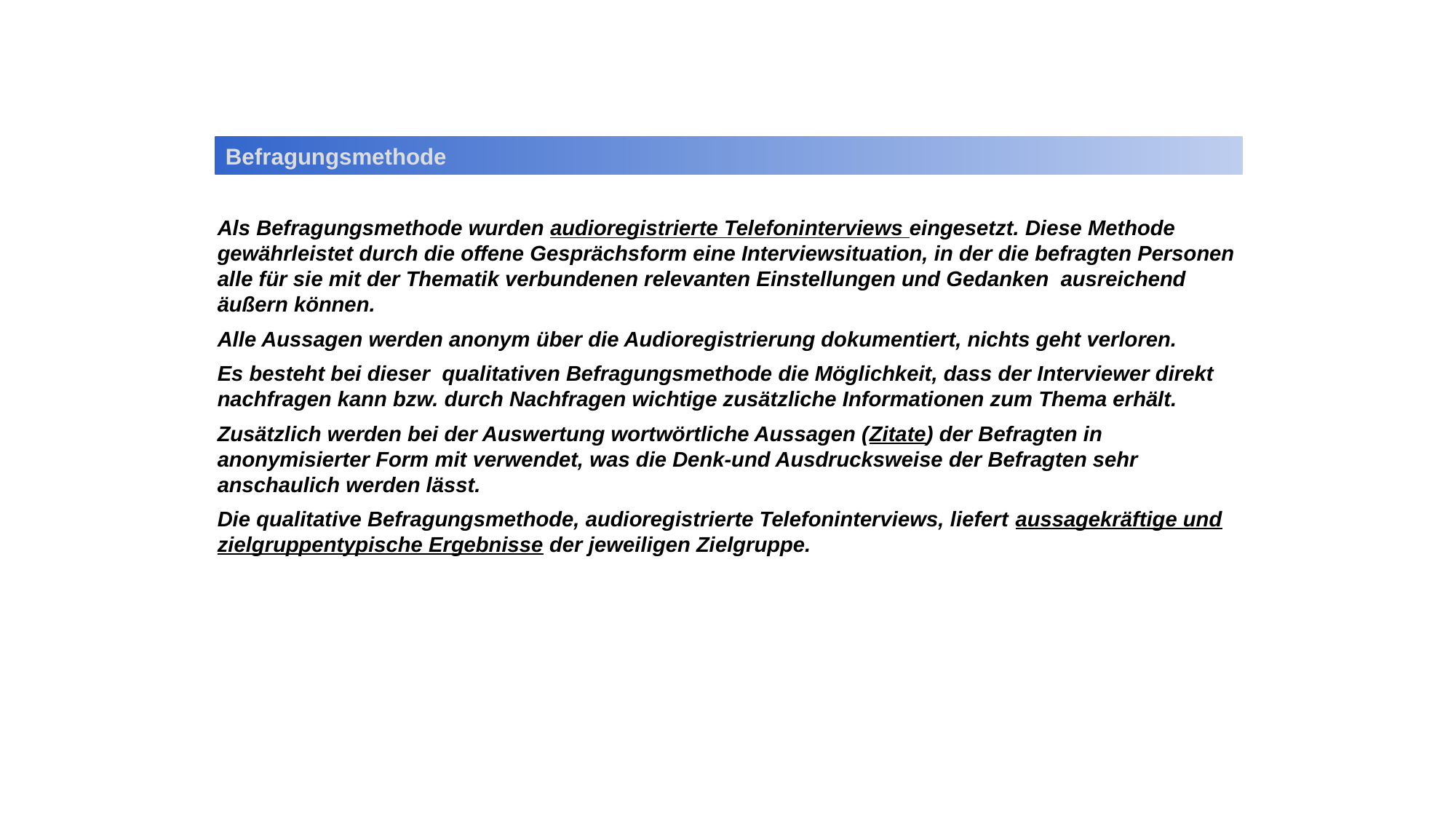

Befragungsmethode
Als Befragungsmethode wurden audioregistrierte Telefoninterviews eingesetzt. Diese Methode gewährleistet durch die offene Gesprächsform eine Interviewsituation, in der die befragten Personen alle für sie mit der Thematik verbundenen relevanten Einstellungen und Gedanken ausreichend äußern können.
Alle Aussagen werden anonym über die Audioregistrierung dokumentiert, nichts geht verloren.
Es besteht bei dieser qualitativen Befragungsmethode die Möglichkeit, dass der Interviewer direkt nachfragen kann bzw. durch Nachfragen wichtige zusätzliche Informationen zum Thema erhält.
Zusätzlich werden bei der Auswertung wortwörtliche Aussagen (Zitate) der Befragten in anonymisierter Form mit verwendet, was die Denk-und Ausdrucksweise der Befragten sehr anschaulich werden lässt.
Die qualitative Befragungsmethode, audioregistrierte Telefoninterviews, liefert aussagekräftige und zielgruppentypische Ergebnisse der jeweiligen Zielgruppe.
