# Supplementary material for: Consequences of the Corona crisis on outpatient oncological care – a qualitative study among nurses and medical assistants
Source: PLoS One. 2022 Oct 21;17(10):e0276573. doi: 10.1371/journal.pone.0276573 (PMC9586350; doi:10.1371/journal.pone.0276573)
Supplement: S1 File — (DOCX) [file pone.0276573.s002.docx]

**Hämatologie Onkologie Palliativmedizin Tagesklinik**

**Belegbetten Krankenhaus Landshut-Achdorf (Akademisches Lehrkrankenhaus der TU München)**

Dr. med. Ursula Vehling-Kaiser • Achdorfer Weg 5 • 84036 Landshut

An die

Ethik-Kommission

der BLÄK

Mühlbaurstraße 16

81677 München

Landshut, 04.05.2020

**Anfrage Ethikvotum: Ambulante Versorgung von Tumorpatienten in der Corona-Krise**

Sehr geehrte Damen und Herren,

wir planen eine retrospektive Befragung zur Versorgungssituation von onkologisch /hämatologischen Patienten im ambulanten Versorgungsbereich in der Zeit der COVID-19 Pandemie. Die Befragung wird in Form audioregistrierter Leitfadeninterviews durchgeführt. Befragt werden Patienten, Ärzte und nichtärztliche Mitarbeiter aus dem onkologisch/palliativmedizinischem Netzwerk Landshut. Bitte teilen Sie uns mit, ob für diese Befragung ein Ethikvotum erforderlich ist.

Für Ihre Bemühungen bedanke ich mich herzlich und verbleibe mit freundlichen Grüßen!

_________________________

Dr. med. Ursula Vehling-Kaiser
