# Supplementary material for: Consequences of the Corona crisis on outpatient oncological care – a qualitative study among nurses and medical assistants
Source: PLoS One. 2022 Oct 21;17(10):e0276573. doi: 10.1371/journal.pone.0276573 (PMC9586350; doi:10.1371/journal.pone.0276573)
Supplement: S2 File — (PDF) [file pone.0276573.s003.pdf]

Ethik-Kommission
der Bayerischen Landesärztekammer

Ethik-Kommission der BLÄK · Mühlbaurstraße 16 · 81677 München

Onkologisches und Palliativmedizinisches
Netzwerk Landshut
Frau Dr. med. Ursula Vehling-Kaiser
Achdorfer Weg 5
84036 Landshut

Telefon: 089 4147-212
Fax: 089 4147-334
E-Mail: ethikkommission@blaek.de

Unser Zeichen: 2020-1109 Dr. AB/Gu
Ihre Zeichen:
Ihr Schreiben vom:

11.05.2020

Anfrage Ethikvotum: Ambulante Versorgung von Tumorpatienten in der Corona-Krise

Unser Zeichen: 2020-1109

Sehr geehrte Frau Dr. Vehling-Kaiser,

wir bestätigen den Eingang Ihres Schreibens vom 05.05.2020 und teilen Ihnen dazu Folgendes mit:

Die retrospektive Befragung zur Versorgungssituation onkologisch/hämatologischer Patienten dient schwerpunktmäßig der Qualitätskontrolle und ist daher nicht beratungspflichtig.

Wir empfehlen jedoch dringend das hinter der Befragung stehende Datenschutzkonzept durch den zuständigen Datenschutzbeauftragten prüfen zu lassen.

Mit freundlichen Grüßen

U. Artmeier-Brandt

Dr. med. Ulrike Artmeier-Brandt
Fachärztin für klinische Pharmakologie und
Anästhesiologie in der Geschäftsführung
der Ethik-Kommission

Die Ethik-Kommission ist bei der BLÄK eingerichtet, § 13a Satzung der Bayerischen Landesärztekammer und Art. 29a GDVG.

Bayerische Landesärztekammer
Körperschaft des öffentlichen Rechts
Mühlbaurstraße 16
81677 München
Telefon 089 4147-0
www.blaek.de

Am besten erreichen Sie die BLÄK telefonisch montags bis donnerstags von 9.00 bis 15.30 Uhr und freitags von 9.00 bis 12.00 Uhr

Bayerische Landesbank München
IBAN DE 19 7005 0000 0000 0248 01
BIC : BYLADEMM
